# Prenatal Screening for Chagas Disease in the Province of Guadalajara, Spain

**DOI:** 10.7759/cureus.62398

**Published:** 2024-06-14

**Authors:** Marta B Roldán Rodríguez, Mario Pérez-Butragueño, Javier E Blanco González, Ramon Perez Tanoira, Alfonso Ortigado Matamala, Alejadro González Praetorius

**Affiliations:** 1 Graduate School, University of Alcala de Henares, Madrid, ESP; 2 Pediatrics, Centro de Salud el Casar, Castilla-La Mancha Health Service (SESCAM), Guadalajara, ESP; 3 Pediatrics, Hospital Universitario Infanta Leonor, Madrid, ESP; 4 Pediatrics, Centro de Salud Azuqueca de Henares, Castilla-La Mancha Health Service (SESCAM), Guadalajara, ESP; 5 Microbiology, Hospital Universitario Príncipe de Asturias, Alcalá de Henares, ESP; 6 Pediatric Cardiology, Hospital Universitario de Guadalajara, Guadalajara, ESP; 7 Microbiology, Hospital Universitario de Guadalajara, Guadalajara, ESP

**Keywords:** neonatal screening, tropical disease, vertical transmission of infectious disease, prenatal diagnosis, chagas disease

## Abstract

Introduction

Chagas disease is caused by the protozoan Trypanosoma cruzi. It is endemic in 21 countries in Central and South America. Spain is the only nonendemic country with the highest number of Chagas disease cases outside the Americas. The only transmission mechanism in Spain is vertical transmission.

Materials and methods

We reviewed the records of pregnant women from endemic countries who underwent prenatal care at the Hospital Universitario de Guadalajara, from January 1, 2009, to December 31, 2022, to determine the rate of Chagas disease screening and vertical transmission.

Results

Out of a total of 1,681 pregnant women from endemic countries, prenatal screening was conducted on 316 (18.7%) of them. According to our study, the prevalence of the disease in the population of pregnant women from endemic countries is 0.95% with a 95% confidence interval (ranging from 0.32% to 2.75%), with three out of the 316 screened women testing positive for the disease. All positive cases were among Bolivian women. Vertical transmission was not observed in any of the cases. However, because of the small sample size, this study cannot conclusively determine the vertical transmission rate in the province of Guadalajara.

Conclusions

Implementing regulated prenatal screening protocols for Chagas disease at regional or national levels is necessary to increase the rate of prenatal screening. Additionally, increasing awareness of this condition among healthcare professionals and at-risk populations could further improve prenatal screening rates and treatment adherence.

## Introduction

Chagas disease is caused by the protozoan Trypanosoma cruzi. It is endemic in 21 countries in Central and South America, with an estimated 6-7 million affected individuals. It is estimated that 9,000 newborns are infected each year during gestation. Between 20% and 30% of those infected will develop cardiac complications, specifically Chagasic cardiomyopathy, which stands as the primary contributor to morbidity and mortality in the disease [1-4].

The predominant mode of transmission in endemic countries is vector-borne, facilitated by hemipterans (bugs) of the Triatominae family. While vertical transmission stands as the primary mechanism of contagion in nonendemic countries. In Spain, it is the only transmission route, as blood and tissue donations have been subjected to Chagas disease screening since 2005, regulated by Real Decreto 1088/2005 [5].

In our setting, the prevalence of Chagas disease in pregnant women is estimated at 3.4% (reaching up to 27.7% in Bolivian pregnant women) [6,7]. The vertical transmission rate is 1-12% depending on the studies [7]. Most pregnant women are usually in the chronic phase of the disease. However, there is a higher risk of preterm birth, low birth weight, or fetal death. Early detection and treatment of infected girls and women of childbearing age (before or after pregnancy), as well as screening of newborns and other children of infected mothers, are considered key by the WHO for disease control and prevention of vertical transmission in subsequent pregnancies [2].

According to the 2015 economic assessment conducted by the Carlos III Health Institute in Spain, it was found that the "no screening" strategy for Chagas disease is both the costliest and the least effective, from both societal and National Health System standpoints. Conversely, screening pregnant women, their newborns, and first- and second-degree relatives of positive mothers emerged as the most efficient screening strategy [8].

In Spain, only three autonomous communities (Catalonia [6], Galicia [9], and the Valencian Community [10]) have already implemented regional prenatal screening programs for Chagas disease in Spain.

The objectives of our study are to determine the rate of prenatal screening for Chagas disease from 2009 to December 31, 2022, in the province of Guadalajara, as well as the rate of vertical transmission during this period.

This article was previously presented as a meeting abstract at the XX Congreso de Actualización en Pediatría organized by the Spanish Association of Primary Care Pediatrics (AEPap) that took place in Madrid from March 7 to 9, 2024.

## Materials and methods

A retrospective study of all pregnant women from endemic countries, including Argentina, Belize, Bolivia, Brazil, Chile, Colombia, Costa Rica, Ecuador, El Salvador, Guatemala, French Guiana, Guyana, Honduras, México, Nicaragua, Panamá, Paraguay, Perú, Suriname, Uruguay, and Venezuela, who received prenatal care at the Hospital Universitario de Guadalajara (Castilla La Mancha-Spain) from January 1, 2009, to December 31, 2022.

The lists of pregnant women from endemic countries who monitored their pregnancy at the Hospital Universitario de Guadalajara during that period were provided by the information technology service of Servicio de Salud de Castilla-La Mancha (SESCAM).

All women from these countries who had at least one routine prenatal serological test during pregnancy were included (regardless of whether the pregnancy was carried to term, resulted in a miscarriage, or was lost to clinical follow-up).

The screening for Chagas disease involved performing a chemiluminescent immunoassay (CLIA) test (VIRCLIA®, Granada, Spain) during the initial serology assessment for pregnant women from endemic regions. VIRCLIA® has reported sensitivity and specificity values of approximately 96-100% and 98-100%, respectively. If the CLIA test result was positive, the samples were referred to the National Microbiology Center for a confirmatory second serological test, typically an "in-house" enzyme-linked immunosorbent assay (ELISA), which confirms the diagnosis of Chagas disease. In cases of discrepancy, a third serological test-usually an "in-house" indirect immunofluorescence assay (IIFA) was performed.

The medical records of newborns born to Chagas-positive mothers were reviewed to determine the rate of vertical transmission.

For newborns born to mothers diagnosed with Chagas disease, the following diagnostic steps were taken. At birth and at one month of age, an “in-house” PCR was performed on peripheral blood by the National Microbiology Center. If the result was negative, between eight and 10 months of age, a serological chemiluminescence test (VIRCLIA®) was conducted. If the result was also negative, Chagas disease was ruled out. If the result was positive, the sample was referred to the National Microbiology Center for a second serological test (usually an "in-house" ELISA) to confirm the diagnosis. In cases of discrepancy, an "in-house" indirect immunofluorescence assay (IIFA) serological test was conducted.

The rate of prevalence of Chagas disease in pregnant women from endemic countries was calculated using the Wilson method.

## Results

**Figure 1:**
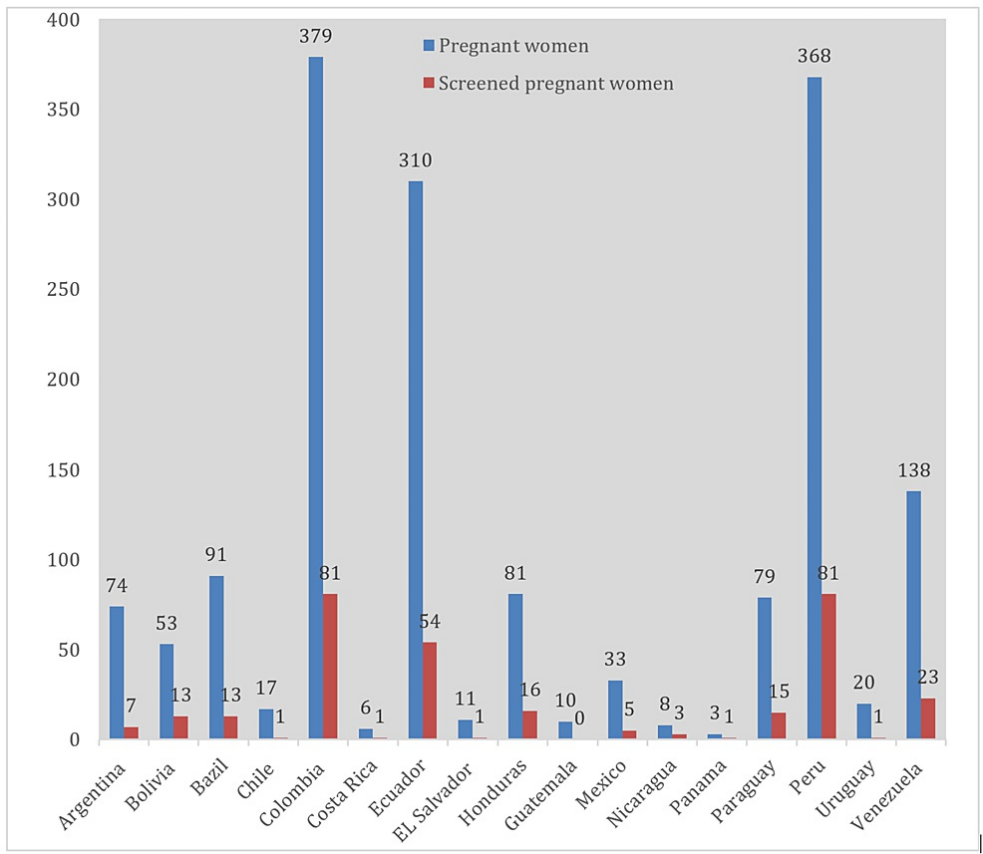
Total number of pregnant women monitored and screened according to the country of origin.

Between January 1, 2009, and December 31, 2022, 1,681 pregnant women, with a total of 2,152 pregnancies, from 17 Chagas-endemic countries, mostly from Colombia, Peru, and Ecuador, received prenatal care at the Hospital Universitario de Guadalajara (Figure 1).

**Figure 2:**
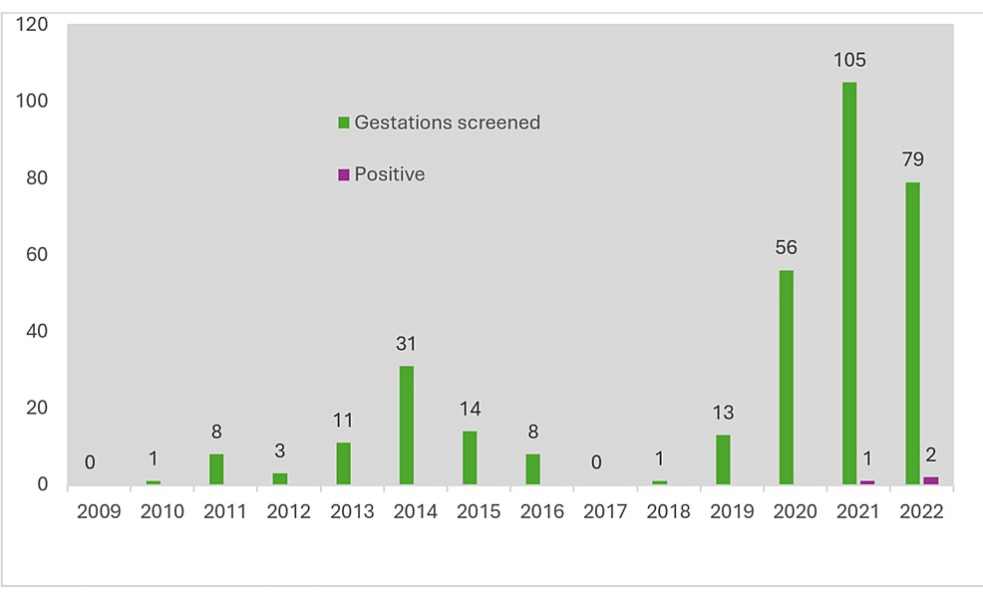
Gestations screened by year.

Serologies have been performed on 316 pregnant women (18.7%), with a total of 330 pregnancies screened. The majority were done in the last three years of the study, with 240 prenatal screenings conducted during this period, representing 38% of the pregnancies monitored in these three years (Figure 2).

The detection rate of Chagas disease in pregnant women from endemic countries is 0.95%, with a 95% confidence interval ranging from 0.32% to 2.75%. In our series, all pregnant women diagnosed with Chagas disease were of Bolivian origin. We found a prevalence of 23.08% with a 95% confidence interval ranging from 8.18% to 50.26% in the Bolivian population. All of them were in the indeterminate chronic phase of the disease at the time of the diagnosis. None of them have received treatment yet, two of them because they are still breastfeeding and the other because she was lost to clinical follow-up.

**Figure 3:**
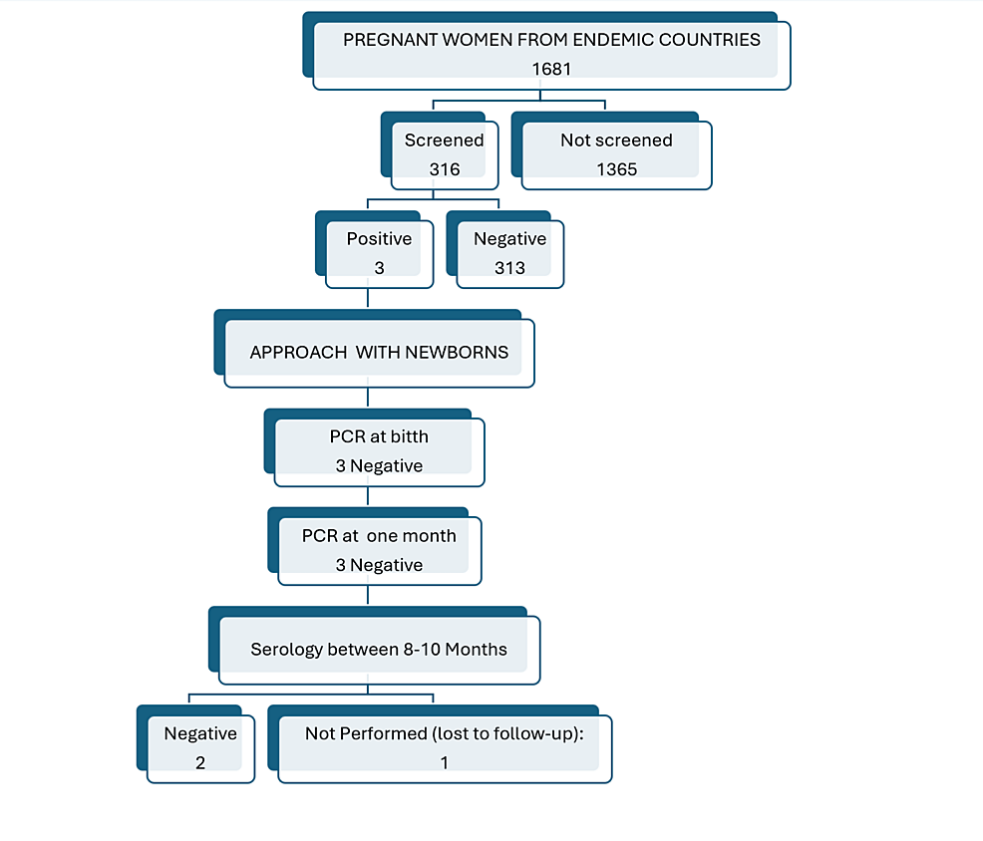
Screening of pregnant women from endemic countries and their newborns.

In the newborns of positive mothers, a PCR test was conducted on peripheral blood at birth and repeated at one month of age. As both tests were negative, serology was performed between eight and 10 months of age for two of the children, which came back negative, ruling out the disease. As for the other child, who is now 10 months old, it would be time to perform the serology now, but he was lost to clinical follow-up (Figure 3). There have been no cases of vertical transmission in our series so far. However, because of the small sample size of only three cases, we cannot draw conclusions about the vertical transmission rate.

## Discussion

Because of migratory movements, in recent times, the presence of patients with Chagas disease has been observed in countries considered nonendemic. Spain is the only nonendemic country with the highest number of Chagas disease cases outside the Americas [1,5]. Currently, in our country, the only transmission mechanism is vertical transmission [5].

The WHO considers that early detection and treatment of infected girls and women of childbearing age (before or after pregnancy), as well as the screening of newborns and other children of infected mothers, is key to controlling the disease and preventing vertical transmission in subsequent pregnancies [2]

Prenatal screening should be performed for every pregnant woman from an endemic country or who has resided in the area for a long period, during the first blood test in the first trimester, along with the other serologies. If this is not possible, it can be done in later stages of pregnancy [6,9,10].

Early diagnosis and treatment of this disease are important because the effectiveness of the treatment to cure the Chagas disease has been shown to be higher if administered during the acute phase, with a cure rate between 65-80%, reaching rates above 95% in cases of congenital transmission. The effectiveness decreases as the infection time progresses, achieving cure rates between 15 and 40% in cases of chronic infection [1-5,11]. Additionally, the treatment does not reverse cardiac involvement once the disease has established itself [12,13]. Moreover, treatment of the mother after childbirth prevents transmission in subsequent pregnancies [4].

In Spain, Catalonia [6], Galicia [9], and the Valencian Community [10] have already implemented regional prenatal screening programs for Chagas disease; however, currently, there is no regulation in the autonomous community of Castilla-La Mancha. However, a prenatal screening program has been implemented in the Hospital Universitario de Guadalajara since 2020 by the microbiology department. Although this program has successfully screened 38.03% of pregnant women from these countries, the involvement of all professionals caring for pregnant women and their newborns is essential to improve prenatal screening rates. Obstetricians, family physicians, primary care pediatricians, and midwives should ensure that prenatal screening is conducted during pregnancy. If not, they should promptly request it. Therefore, the creation of regulated prenatal screening protocols for Chagas disease at a regional or even national level involving all these professionals is necessary to achieve this goal.

**Figure 4:**
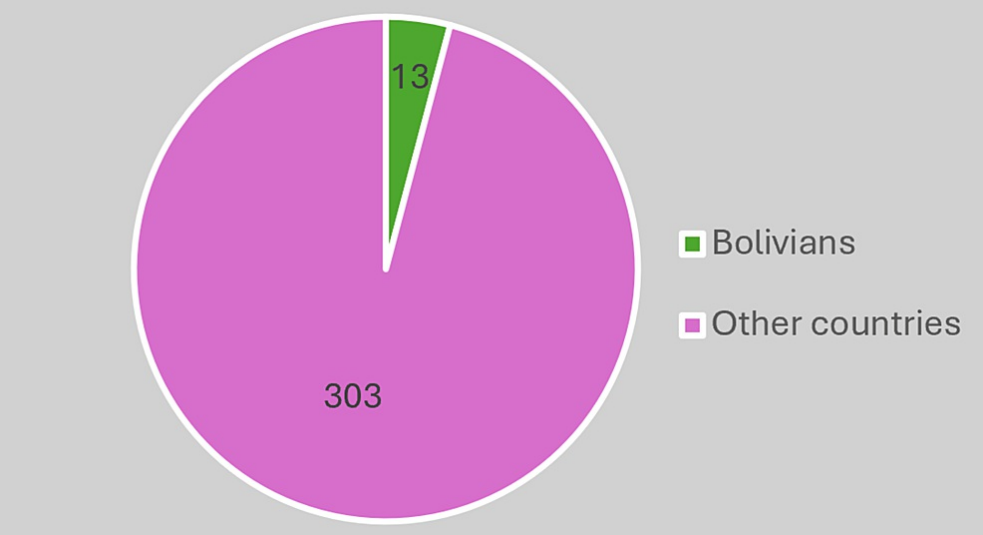
Screened pregnant women.

In our series, all three pregnant women diagnosed with Chagas disease were of Bolivian origin, despite Bolivian pregnant women representing only 3.15% of those monitored in this period (Figure 4).

**Figure 5:**
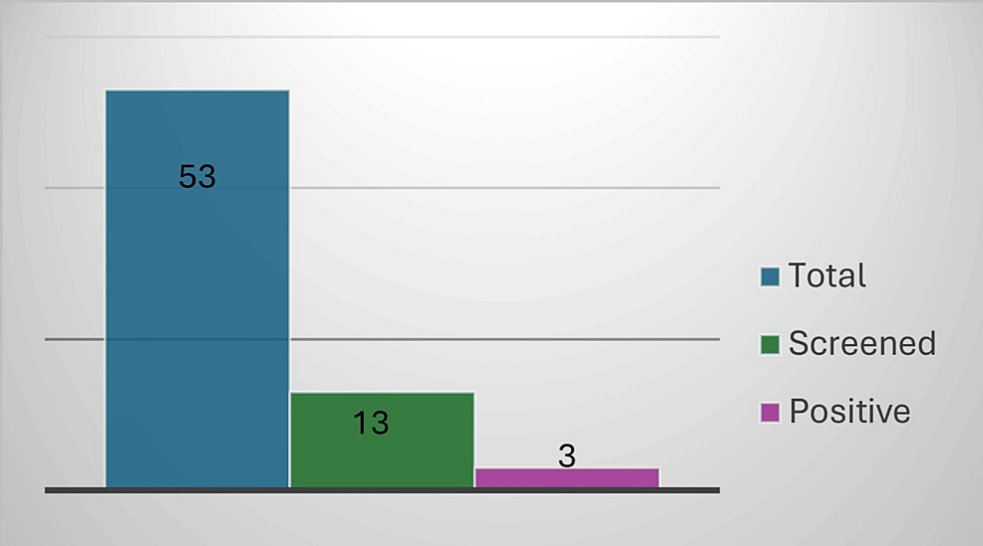
Bolivian pregnant women.

Although Bolivia has the highest prevalence, screening was only conducted on 24.52% of pregnant women from this country (Figure 5).

**Figure 6:**
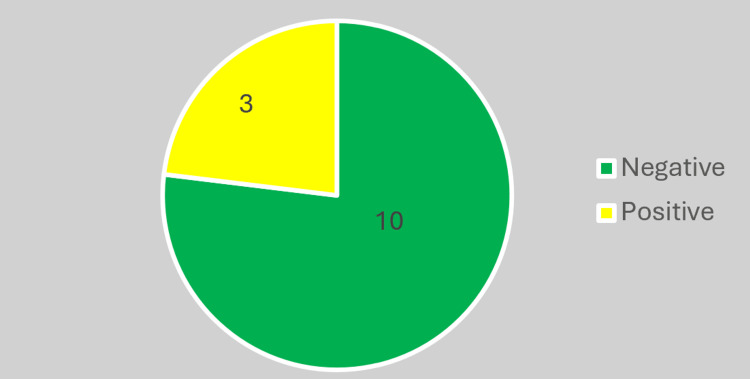
Screened Bolivian pregnant women.

The prevalence among screened Bolivian pregnant women, in our series, was 23.07% with a 95% confidence interval ranging from 8.18% to 50.26%, which is not significantly different from the 27.7% reported in larger series [14] (Figure 6). Therefore, while it is important to screen all pregnant women from endemic countries, special emphasis should be placed on those of Bolivian origin.

Notably, the prevalence of Chagas disease in our series is 0.95% with a 95% confidence interval ranging from 0.32% to 2.75%, below the prevalence found in other studies conducted in our setting [6,7], possibly because of the low number of Bolivian pregnant women in our population.

None of the three women with positive results have received treatment yet. These data align with the low treatment rate of diagnosed Chagas disease patients reported in the literature, where less than 15% of diagnosed patients receive treatment [14,15]. This may be attributed to a lack of awareness about the seriousness of this chronic disease, which often presents asymptomatically. Therefore, it would be advisable to focus on awareness campaigns for this disease.

We have not detected any cases of vertical transmission in our series. However, because of the small sample size, this study cannot conclude that the vertical transmission rate in the province of Guadalajara is 0%. It is essential to increase the prenatal screening rate for Chagas disease to draw conclusions about the vertical transmission rate that can be generalized to the entire migrant population in the province of Guadalajara.

The main limitation of this study is the low prenatal screening rate because of the lack of an official universal screening program involving all healthcare professionals attending to these women. As a result, we cannot draw conclusions about the vertical transmission rate or the follow-up and management of newborns affected by Chagas disease.

In April 2024, a "Consensus Document for the Prenatal Screening of Chagas Disease" was approved within the National Health System’s Prenatal Screening Program [16]. We hope that this will lead to an increase in the prenatal screening rate for this disease across the entire national territory.

## Conclusions

In our setting, because of the lack of official regional or national protocols for the prenatal screening of Chagas disease, there is a low screening rate. It is crucial to regulate the screening for Chagas disease in pregnant women at a regional or even national level to improve the prenatal screening rate. To achieve this goal, it is also essential to engage all professionals involved in the care of pregnant women and their newborns to ensure early diagnosis and treatment of this disease, preventing vertical transmission in future pregnancies and medium to long-term target organ damage in these patients.

Furthermore, we believe that increased awareness of this disease among healthcare professionals and at-risk populations would improve the rate of prenatal screening and increase treatment adherence.
